# Mitochondrial Cholesterol Metabolites in a Bile Acid Synthetic Pathway Drive Nonalcoholic Fatty Liver Disease: A Revised “Two-Hit” Hypothesis

**DOI:** 10.3390/cells12101434

**Published:** 2023-05-20

**Authors:** Genta Kakiyama, Daniel Rodriguez-Agudo, William M. Pandak

**Affiliations:** 1 Department of Internal Medicine, Virginia Commonwealth University School of Medicine, Richmond, VA 23298, USA; daniel.rodriguezagudo@vcuhealth.org (D.R.-A.); william.pandakjr@vcuhealth.org (W.M.P.); 2 Research Services, Central Virginia Veterans Affairs Healthcare System, Richmond, VA 23249, USA

**Keywords:** bile acid pathways, cholesterol metabolism, hepatotoxicity, mitochondria, oxysterols, insulin resistance, fatty liver disease

## Abstract

The rising prevalence of nonalcoholic fatty liver disease (NAFLD)-related cirrhosis highlights the need for a better understanding of the molecular mechanisms responsible for driving the transition of hepatic steatosis (fatty liver; NAFL) to steatohepatitis (NASH) and fibrosis/cirrhosis. Obesity-related insulin resistance (IR) is a well-known hallmark of early NAFLD progression, yet the mechanism linking aberrant insulin signaling to hepatocyte inflammation has remained unclear. Recently, as a function of more distinctly defining the regulation of mechanistic pathways, hepatocyte toxicity as mediated by hepatic free cholesterol and its metabolites has emerged as fundamental to the subsequent necroinflammation/fibrosis characteristics of NASH. More specifically, aberrant hepatocyte insulin signaling, as found with IR, leads to dysregulation in bile acid biosynthetic pathways with the subsequent intracellular accumulation of mitochondrial CYP27A1-derived cholesterol metabolites, (*25R*)26-hydroxycholesterol and 3β-Hydroxy-5-cholesten-(*25R*)26-oic acid, which appear to be responsible for driving hepatocyte toxicity. These findings bring forth a “two-hit” interpretation as to how NAFL progresses to NAFLD: abnormal hepatocyte insulin signaling, as occurs with IR, develops as a “first hit” that sequentially drives the accumulation of toxic CYP27A1-driven cholesterol metabolites as the “second hit”. In the following review, we examine the mechanistic pathway by which mitochondria-derived cholesterol metabolites drive the development of NASH. Insights into mechanistic approaches for effective NASH intervention are provided.

## 1. Introduction

Nonalcoholic fatty liver disease (NAFLD) was first reported in 1980 [1]. In the four decades since, its prevalence has dramatically increased. Currently, NAFLD affects an estimated 25% of the global adult population and is the most common chronic liver disease worldwide [2,3]. NAFLD is currently the second leading indication for liver transplantation in the United States and will likely become the number-one indication in the near future. NAFLD is a metabolic disorder closely associated with obesity and type 2 diabetes mellitus (T2DM); it is prevalent in T2DM patients with a global incidence of 55% [4]. However, the correlation of NAFLD with T2DM is markedly higher when insulin resistance (IR) is used as the true definition of T2DM. In recent clinical trials in patients with NAFLD [5,6,7,8,9,10,11], improving insulin sensitization using diabetes treating agents (i.e., a GLP-1 agonist) was shown to improve liver histology in NAFLD. Even though the clinical data suggest that IR plays a pivotal role in NAFLD development, the direct causative factors for the development and progression of NAFLD remain unclear. To achieve optimal clinical results, a complete “mechanistic” understanding of the metabolic pathways that initiate and perpetuate the transition of steatosis (or fatty liver; NAFL) to steatohepatitis (NASH) is necessary. Simply put, a limited understanding of disease etiology limits the therapeutic disease-modifying options for patients with NAFLD. Currently, there are no FDA approved medications for the treatment of NAFLD [12].

**Figure 1:**
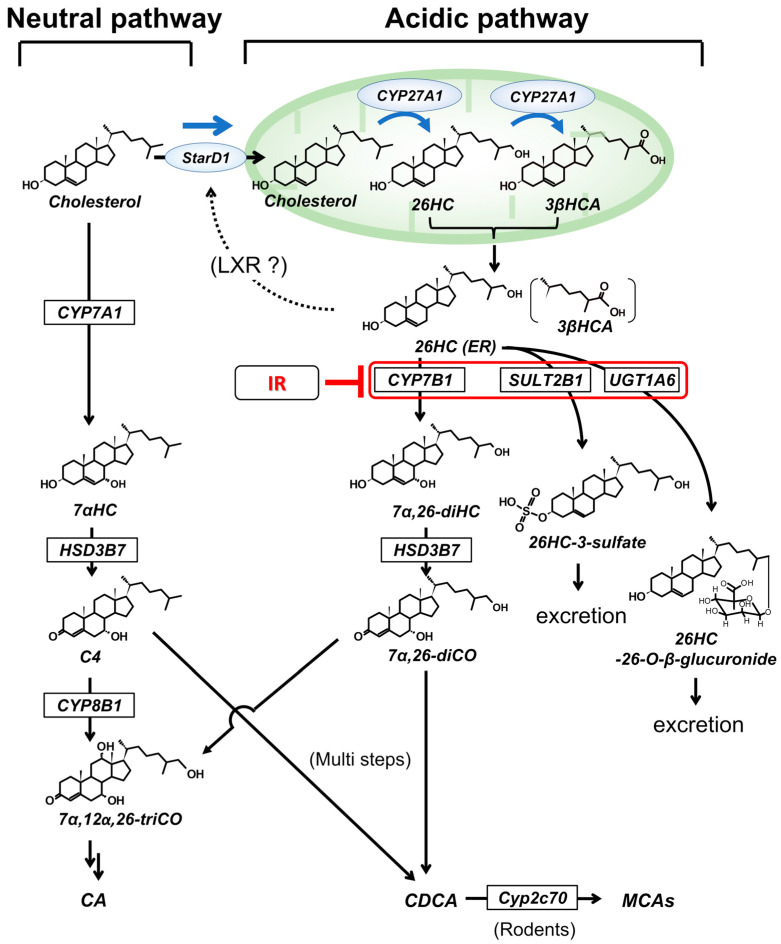
Major cholesterol metabolic pathways to bile acids in the liver—IR drives mitochondrial cholesterol accumulation. StarD1 carries cholesterol into the inner mitochondrial membrane (IMM), where CYP27A1 hydroxylates a cholesterol side chain to generate 26HC. Part of 26HC can be further oxidized to 3βHCA when excess 26HC is present. An ER-resident CYP7B1 7α-hydroxylates 26HC (and 3βHCA: not shown in this illustration) to form 7α,26-diHC (3β,7α-diHCA), facilitating their conversion to CDCA (and MCAs in mice). Subsequent HSD3B7 (3βHSD) converts 7α,26-diHC into 7α,26-diHCO prior to the reduction of the Δ^4^-bond. Mitochondrial 26HC and 3βHCA can be metabolized by glucuronidation and sulfation as well. CYP7A1, the rate-determining enzyme of the neutral pathway whose expression, like that of CYP7B1, UGT1A6, and SULT2B1, is also regulated by insulin signaling. Impaired CYP7B1 expression in the setting of IR or in its genetic deficiency creates a described environment for the toxic accumulation of 26HC/3βHCA in hepatocyte mitochondria. Meanwhile, genetic HSD3B7 deficiency leads to the accumulation of CYP7B1 metabolites (i.e., 7α,26-diHC). However, it is associated with a milder cholestasis, providing evidence that CYP7B1 activity plays a greater role in lessening the toxic effects of 26HC/3βHCA. IR impairs Ugt1a6 and Sult2b1 expression, potentiating the accumulation of 26HC/3βHCA. StarD1 is tightly regulated to prevent mitochondrial cholesterol overload to generate excess 26HC/3βHCA, averting 26HC/3βHCA-driven hepatocyte toxicity. Conversely, StarD1 protein expression appears increased under IR conditions, facilitating cholesterol transport into IMM and furthering 26HC/3βHCA synthesis, overwhelming the compensatory sulfation/glucuronidation of 26HC and 3βHCA. Although not clearly defined, accumulating 26HC and 3βHCA appear to upregulate StarD1 in a feed-forward manner [20,25]. The correlation of StarD1 protein expression with 26HC/3βHCA levels and hepatic toxicity suggests its level of expression plays an underappreciated role in controlling levels of mitochondrial cholesterol metabolites. Abbreviations: CA, cholic acid; CDCA, chenodeoxycholic acid; C4, 3-Oxocholest-4-en-7α-ol; CYP7A1, cholesterol 7α-hydroxylase; CYP7B1, oxysterol 7α-hydroxylase; CYP27A1, sterol 26-hydroxylase; HSD3B7 (3βHSD), 3β-hydroxy-Δ^5^-C27-steroid dehydrogenase/isomerase; IMM, inner mitochondrial membrane; MCA, muricholic acid; StarD1, steroidogenic acute regulatory protein, SULT2B1 (Sult2b1 donates murine), sulfotransferase 2b1; UGT1A6 (Ugt1a6 donated murine), UDP-glucuronosyl transferase 1A6; 3βHCA, 3β-hydroxy-5-cholesten-(*25R*)26-oic acid; 7α-HC, 7α-hydroxycholesterol; 7α,26-diHC, 7α,(*25R*)26-dihydroxycholesterol; 7α,26-diHCO, 3-Oxocholest-4-en-7α,(*25R*)26-diol; 26HC, (*25R*)26-hydroxycholesterol; 26HC3S, (*25R*)26-hydroxycholesterol-3-sulfate; 26HC-26-*O*-GlcA, 26-hydroxycholesterol-(*25R*)26-*O*-β-glucuronide.

It is now well-appreciated that the dysregulation of bile acid homeostasis is found in the metabolic diseases T2DM and NAFLD [13,14,15]. In addition, hepatic free cholesterol and cholesterol-metabolite-mediated toxicity have emerged as likely mechanistic drivers for the necroinflammation and fibrosis characteristic of NASH [16]. In the liver, the metabolism of cholesterol into bile acid mainly occurs via two tightly regulated pathways [14]: the microsomal CYP7A1-initiated “neutral (classical) pathway” and the mitochondrial CYP27A1-initiated “acidic (alternative) pathway” (Figure 1). The neutral pathway is the predominant producer of bile acids, while the foremost role of the acidic pathway is to generate oxysterols (i.e., (*25R*)26-hydroxycholesterol (26HC), 25-hydroxycholesterol (25HC), and 24(*S*)-hydroxycholesterol (24HC)) and cholestenoic acids (i.e., 3β-hydroxy-5-cholesten-(*25R*)26-oic acid (3βHCA)) [17] that control cellular cholesterol and lipid homeostasis [14,18,19]. Our recent findings indicate a key enzymatic step in the acidic pathway to be regulated by both insulin and glucagon [20,21,22]. These observations prompted us to hypothesize that IR in NAFLD might be linked to the dysregulation of cholesterol metabolism via the acidic pathway [14]. Specifically, it was hypothesized that insufficient insulin signaling could lead to the toxic accumulation of mitochondrial CYP27A1-driven cholesterol metabolites such as 26HC and 3βHCA within hepatocytes that trigger subsequent toxic/inflammatory pathways. Of note, in the following discussion, we use the term “IR” for the general pathological condition describing a reduced hepatocyte response to insulin and/or a paucity of hepatocellular insulin. The cellular/molecular mechanism of how IR is developed in NAFLD [23,24] is not the scope of the current review.

In this review, we examine the existing literature to determine if liver-mitochondria-driven cholesterol metabolites are key pathogenetic factors driving fatty liver to inflammation (NASH). We propose a mechanistically derived “two-hit hypothesis” describing how IR-mediated dysregulation drives the increase and chronic accumulation of mitochondria-driven cholesterol metabolites (i.e., oxysterols and cholestenoic acids) that result in the development of NASH, with IR developing as the “first hit” that subsequently leads to the accumulation of toxic cholesterol metabolites as the “second hit”. The current review will provide a renewed mechanistic insight into understanding the pathogenesis of NAFLD and provide an insight into new therapeutic approaches.

## 2. Hepatic Cholesterol Metabolism

The liver is considered the most important organ controlling the body’s cholesterol and lipid homeostasis. Under normal physiologic conditions, the liver tissue maintains relatively low amounts of cholesterol yet deals with a high flow of sterols, consistent with its role in the synthesis and homeostasis of lipoproteins and bile acid [26]. Hepatic bile acid synthesis accounts for a major fraction of daily cholesterol turnover. Under physiological conditions, the metabolism of cholesterol into bile acids occurs via two main pathways (Figure 1). In humans, the neutral (or classical) pathway accounts for most bile acid production (>90%), whereas the acidic (or alternative) pathway accounts for much a smaller portion (up to 10%). The neutral pathway is initiated by a liver-specific cytochrome P-450, cholesterol 7α-hydroxylase (CYP7A1). The product, 7α-Hydroxycholesterol (7αHC), is then rapidly converted into 7α-hydroxy-4-cholesten-3-one (often called C4) by the action of 3β-hydroxy-Δ^5^-C27-steroid dehydrogenase/isomerase (3βHSD; HSD3B7). The subsequent microsomal sterol 12α-hydroxylase (CYP8B1) determines the ratio of cholic acid (CA: 3α,7α,12α-trihydroxy-5β-cholanoic acid) to chenodeoxycholic acid (CDCA: 3α,7α-dihydroxy-5β-cholanoic acid). Of note, in rodents, CDCA is rapidly metabolized to muricholic acids (3α,6ξ,7α-dihydroxy-5β-cholanoic acid) by Cyp2c70 [27]; therefore, little CDCA is present in mouse gallbladder bile [28]. 

The alternative (acidic) pathway starts with the transport of cholesterol into the inner mitochondrial membrane (IMM), where sterol 26-hydroxylase (CYP27A1) is present. Most mitochondrial cholesterol transport is accomplished through the lipid transfer proteins at membrane contact sites or cytosolic diffusible lipid proteins [29]. Steroidogenic acute regulatory protein (StarD1) has been identified as the predominant cholesterol carrier from outer mitochondrial membrane (OMM) to the IMM [30,31]. Global StarD1 knockout mice develop congenital lipoid hyperplasia, and all mice die within 10 days after birth [32]. This indicates that other lipid transporters (i.e., MLN64 [33,34]) do not compensate for StarD1 function. CYP27A1 is constitutively expressed in the IMM. Once cholesterol is transported to the IMM, CYP27A1 immediately hydroxylates cholesterol to form (*25R*)26-hydroxycholesterol (26HC) [17,35]. Since 26HC has a terminal hydroxy group, part of 26HC can be further hydroxylated into a carboxylic acid, 3βHCA, by CYP27A1. Which metabolic conditions lead 26HC to 3βHCA is unknown [36,37,38]. Both 26HC and 3βHCA are regulatory molecules that bind to LXRα in vitro in human hepatocytes [19] and promote reverse cholesterol transport [39,40,41]. Additionally, 26HC and 3βHCA can bind to sterol regulatory element-binding protein (SREBP)-2 [42], which leads to the downregulation of hydroxy-methylglutaryl-CoA reductase (HMGCR), inhibiting de novo cholesterol synthesis. However, the true functions of 26HC and 3βHCA in vivo are still being debated [43]. It is of importance that unlike hepatocyte-specific CYP7A1, CYP27A1 is widely expressed in different organs and cells. A mutation in CYP27A1 disrupts the side-chain oxidation of cholesterol, which is a necessary step for subsequent side-chain shortening reactions for bile acid synthesis. Therefore, patients with a CYP27A1 deficiency develop cerebrotendinous xanthomatosis (CTX), which is a devastating neurological milieu, due to the accumulation of cholesterol and 5α-cholestanol in the brain and tendons [44,45]. It is particularly interesting that although there are several reports of CYP27A1 deficiency causing neonatal cholestasis [46,47,48,49], most adult CTX patients have normal liver function despite their high levels of cholesterol and 5α-cholestanol [50,51]. 

In healthy liver, microsomal oxysterol 7α-hydroxylase (CYP7B1) quickly hydroxylates 26HC and 3βHCA, reducing their regulatory and cytotoxic properties. Human *CYP7B1* cDNA shares 40% of its sequence identity with *CYP7A1* [52]. This enzyme specifically acts on 25HC, 26HC, 3βHCA, and some steroids (i.e., pregnenolone and dehydroepiandrosterone) in steroidogenic tissues [53]. It does not act on endogenous cholesterol. Subsequently, HSD3B7 converts 7α-hydroxylated metabolites (7α,(*25R*)26-dihydroxycholesterol (7α,26-diHC) and 3α,7α-dihydroxy-5-cholesten-(*25R*)26-oic acid (not shown in the figure)) to their 3-Oxo-Δ^4^ forms (7α,(*25R*)26-dihydroxy-4-cholesten-3-one (7α,26-diCO); 3-Oxo-7α-hydroxy-4-cholesten-(*25R*)26-oic acid (not shown in the figure)). These intermediary sterols (or acids) are predominantly converted into CDCA (MCA in rodents). However, there is evidence that 26HC and 3βHCA can also be converted into CA in rodent hepatocytes [54]. It should be emphasized that the dysfunction of CYP7B1 disrupts the metabolism of 26HC and 3βHCA, leading to the chronic accumulation of a developing toxic milieu. Therefore, neonates with the CYP7B1 deficiency rapidly reveal hepatic inflammation that progresses to severe fibrosis and cirrhosis [55,56,57] when exposed to the chronic accumulation of high levels of mitochondria-derived cholesterol metabolites. Supportive of this understanding are patients with HSD3B7 deficiency, who predominantly accumulate their 7α-hydroxylated metabolites (i.e., 3β,7α-diHCA), revealing much milder cholestasis [58,59]. These observations, coupled with those in CTX patients, suggest that specific mitochondria-driven cholesterol metabolites are the key molecules causing hepatic toxicity. With this in mind, it was initially questioned why Cyp7b1 knockout (Cyp7b1^−/−^) mice have normal liver function [22,60], unlike in human cases of CYP7B1 deficiency. However, differing from humans, Cyp7b1^−/−^ mice can alternatively use Sult2b1 and Ugt1a6a for the detoxification of 26HC and 3βHCA. In addition, StarD1 was diminished in the Cyp7b1^−/−^ mouse livers [61]. This ability of the Cyp7b1^−/−^ mouse liver to maintain cellular oxysterols and cholestenoic acid presents new strategies for NASH intervention which will be discussed later.

## 3. Key Concept of the Two-Hit Hypothesis

Succinctly put, a dysregulated mitochondrial cholesterol metabolism, as found with IR, can be hepatotoxic, generating the subsequent inflammatory response found in NASH. The key to understanding the transition from NAFL to NASH centers on the early “initiating” events, which were not described in the literature until recently. Table 1 lists the representative literature that mechanistically studied the role of mitochondria-driven cholesterol metabolites in the development of NASH. Depending on the study approach, including animal models, NAFLD stage, patient cohort, or sex differences [62], contradictory results have been reported in the role of cholesterol metabolites in the pathogenesis of NAFLD and for that matter, the role of lipids. Furthermore, the definition of where NAFLD is first apparent is not clearly defined, with many not considering early elevation in plasma aspartate aminotransferase (AST) and alanine transferase (ALT) levels to be evidence of early toxicity. Just as importantly, many models are designed to study “well-established” NAFLD in which overlapping compensatory inflammation, cell stress, organelle toxicity, etc., begin to occur in addition to the underlying condition, causing the understanding of what initiates the transition to be lost. It is also noteworthy that non-physiologic animal models have been widely used which lose the important pathological features of NAFLD, such as IR [63]. The fact that cholesterol must be added to diets to develop NAFLD has been minimized in the face of the high lipid content that is generally present, and the essential sequential reason for the presence of both is underappreciated. Therefore, careful comparison is needed when examining the existing literature as to whether the data support the proposed two-hit hypothesis.

## 4. Insulin Regulation of Mitochondrial Bile Acid Intermediates

Tissue oxysterol levels are tightly regulated by their synthesis (i.e., StarD1; CYP27A1) and metabolism (i.e., CYP7B1), which are coordinately regulated by various factors such as insulin, cytokines, bile acids, and hormones. Moreover, the ability of hydroxysteroid sulfotransferase (SULT) and UDT-glucuronosyl transferase (UGT) to metabolite oxysterols represent additional means through which to metabolically regulate cellular levels of cholesterol and oxysterols, including 25HC and 26HC [21]. The potential importance of the insulin-mediated coordinated regulation of these genes has never been truly addressed in the context of NAFLD.

Insulin regulates Cyp7b1 through hepatocyte nuclear factor (HNF)-4α [69,70]. HNF4α is one of the most abundant transcription factors in the liver and is a well-described regulator of glucose metabolism, lipid metabolism, and insulin secretion. HNF4A gene mutation causes maturity-onset diabetes of the young type 1 (MODY1), which is associated with an increased risk of T2DM [71]. Therefore, any disruption in this signaling pathway can lead to Cyp7b1 disruption, resulting in impaired 26HC and 3βHCA metabolism and a developing hepatotoxic milieu. In obese, IR, or DM mouse models, Cyp7b1 is significantly downregulated [72,73]. Biddinger et al., reported that hepatocyte insulin receptor deletion led to significantly reduced *Cyp7b1* mRNA in mouse livers [74]. Conversely, Tang et al. demonstrated that the upregulation of the insulin signaling pathway seemed able to restore Cyp7b1 [75]. Our group has also provided evidence of impaired hepatic Cyp7b1 in multiple mouse models of IR, including WD-induced NAFL mice (B6/129) and streptozotocin (STZ)-treated diabetic mice [20,21]. Moreover, it is reported Sult2b1 is regulated by Hnf4α [21,76]. Therefore, the dysregulation of the insulin-Hnf4α-Cyp7b1/Sult2b1 pathway can contribute to 26HC/3βHCA accumulation.

Meanwhile, cholesterol transport to the IMM is a fundamental step of mitochondrial 26HC/3βHCA synthesis [77,78]. Cholesterol trafficking from the OMM to the IMM is mediated by a highly regulated multimeric protein complex comprising StarD1, mitochondrial translocator protein, a voltage-dependent anion channel, adenine nucleotide transporter, and associated regulatory proteins [29]. Little is known if insulin directly regulates this system. However, it appears that in NAFLD, hepatic StarD1 is upregulated both in human [79] and in mouse models [22]. Alternatively, StarD1 appears to be positively regulated by LXRα [25]. If this is the case, lower hepatocellular insulin responsiveness due to developing IR leads to suppressed levels of Cyp7b1/Sult2b1, with resultant increases in mitochondrial 26HC/3βHCA. In a feed-forward manner, chronic persistent increases in hepatocellular 26HC upregulate StarD1 [14,20,25], leading to further cell accumulation of 26HC/3βHCA (Figure 1). However, it should be mentioned that Saito et al. very recently reported that endogenous 26HC failed to induce LXRα target gene expressions in a primary rat hepatocyte model [42]. Further investigation is clearly needed into the StarD1 induction mechanism in fatty liver. Of note, as with Cyp7b1 and Sult2b1, Cyp27a1 gene transcription is regulated by Hnf4α [80]. It is possible that a paucity of insulin signaling (IR) may lead to a reduction in Cyp27a1, thereby reducing the formation of 26HC/3βHCA. However, we and others have found Cyp27a1 to be mostly constitutively regulated, a rationale for this effect appearing to be minimal [61]. In support of this statement, when compared to StarD1 overexpression, we found little increase in the synthesis of 26HC with the overexpression of Cyp27a1 in HepG2 and primary rodent hepatocyte cultures [30,81]. Therefore, the formation of 26HC/3βHCA relies more on the supplementation of a cholesterol substrate into the IMM rather than Cyp27a1 expression levels [31].

## 5. Cholesterol Metabolites in Mitochondria Impairment 

Dysregulation of the mitochondrial cholesterol metabolism and the resulting lipotoxicity have been identified in many metabolic diseases such as cancer, heart, and liver diseases [79,80]. Elevated hepatic StarD1 is reported in patients with steatosis and NASH [79] or NASH-driven HCC [82]. Elevated cholesterol contents in liver mitochondria have also been demonstrated in experimental NASH models [77,83,84]. Mitochondrial cholesterol accumulation can cause reduced mitochondrial membrane fluidity [85], reduced ATP generation [86,87], impaired mitochondrial glutathione import [83,88,89,90] and oxidative phosphorylation, and the disrupted assembly of respiratory super complexes [91]. Very recently, our group also demonstrated in a WD-induced fatty liver mouse model that the accumulation of 26HC/3βHCA in combination with cholesterol in the liver mitochondria, can lead to downregulations in oxidative-phosphorylation-related mRNAs such as mitochondrial encoded units of the electron transport chain, including NADH dehydrogenase in Complex I (i.e., NADH dehydrogenase 1-4: *mt.Nd1-4*), cytochrome c reductase in complex III (i.e., mitochondrially encoded cytochrome b: *mt.Cytb*), cytochrome c oxidase in Complex IV (i.e., mitochondrially encoded cytochrome c oxidase II: *mt.Co2*), and F- and V-type ATPase (i.e., mitochondrially encoded ATP synthase 6: *mt.Atp6*) in Complex V [22]. 

A question arises: “Would mitochondrial dysfunction be led by cholesterol itself or by Cyp27a1-mediated metabolites with early NAFLD conditions?” With elevated mitochondrial cholesterol transport in fatty liver, IMM Cyp27a1 immediately converts cholesterol into 26HC and 3βHCA. Perhaps this question might be answered by observations in models of Cyp27a1 deficiency. Although not in all cases, most adult CTX patients have normal liver function despite their high cholesterol and 5α-cholestanol [50,51]. Similarly, in an atherogenic mouse model with Cyp27a1 deficiency, Zurkinden et al. [92,93] reported that Cyp27a1/ApoE double-knockout mice were resistant to WD-induced liver inflammation, as demonstrated by their lowered hepatic inflammatory and oxidative stress gene expressions (i.e., Tnfα and Il1b). Another study in a goose model reported that hepatic *Cyp27a1* mRNA expression can be significantly inhibited in goose fatty livers [94]. Geese are known to accumulate significant amounts of fat before migration, but their livers do not reveal any NASH-like pathology such as hepatic inflammation or fibrosis. Whether 26HC or 3βHCA relate to the mitochondrial dysfunction remained unaddressed (which is challenging to study in vivo); these observations signify that mitochondrial CYP27A1 activity is necessary to drive mitochondrial toxicity and subsequent hepatocellular inflammation/fibrosis.

It should be mentioned that when combined with cholesterol in hepatocytes, fatty acids can undergo lipid peroxidation to form nonenzymatic oxysterols that cause the impairment of mitochondrial function, biogenesis, and the depletion of respiratory chain complexes in NAFLD [95,96]. Exposing the hepatocyte to the combination of fatty acids (palmitic and oleic acids) and an oxysterol, cholestane-3β,5α,6β-triol, can cause mitochondrial dysfunction [97,98]. However, these events may represent a more chronic state. To the best of our knowledge, there are no data on the synergistic interaction of free fatty acids and CYP27A1-driven oxysterols (26HC/3βHCA) causing mitochondrial dysbiogenesis. To identify the exact molecule, the mechanism driving mitochondrial dysfunction in the metabolic conditions of early IR and the characterization of oxysterol (fatty acid) content in mitochondrial lipid raft domains would aid in understanding how oxysterols alter the structure and function of the mitochondrial membrane.

It should also be noted that the exact mechanism of how the 26HC and 3βHCA within the mitochondria of hepatocytes trigger the inflammatory response in immune cells remains unclear. In mice with accumulated mitochondrial 26HC/3βHCA in the early stages of NAFLD, hepatic genes involved in oxidative phosphorylation, particularly the genes encoded by mitochondrial DNA (mtDNA), were downregulated [22]. This observation suggests decreased transcription or damaged mitochondrial DNA. As reviewed by Zhang et al. [99], increased hepatocyte mitochondrial stress leads to an increase in the release of damaged or fragmented mtDNA into cytosol through the mitochondrial permeability transition pore. Hepatic non-parenchymal cells, including neutrophils, Kupffer cells, and dendritic cells, can take free mtDNA and mtDNA-enriched microparticles, thereby triggering the inflammatory response. Recently, Yu et al. demonstrated that mtDNA from the hepatocytes of mice fed a high-fat diet led to increased levels of tumor necrosis factor (Tnf)α and interleukin (Il)-6 expression in co-cultured Kupffer cells, demonstrating its ability to activate the nuclear factor-κB (NF-κB) signaling pathway [100]. The activation of the NF-kB pathway by 26HC was also reported in endothelial cells [101]. Further study using the early fatty liver model is needed for clarifying a more detailed mechanism. 

## 6. Cholesterol Metabolites and Endoplasmic Reticulum Function 

Endoplasmic reticulum (ER) stress is another prevailing theory of driving NAFLD to NASH [102]. As reviewed by Horn et al. [16], elevated levels of free cholesterol in the ER membrane can impair the action of sarco/ER Ca^2+^-ATPase (SERCA) in mice, a pump that maintains a high Ca^2+^ concentration in the ER lumen to facilitate protein folding [103,104,105,106]. The impaired functionality of SERCA results in a decrease in luminal calcium concentration, higher levels of unfolded proteins, and ER stress. Activation of the unfolded protein response (UPR) leads to the upregulation of key enzymes that alleviate ER stress by decreasing the ER secretory load and enhancing protein folding. However, our recent studies observed that no ER-stress-related pathways were induced in a WD-induced early NAFL mouse model despite the hepatic accumulation of cholesterol and Cyp27a1-driven cholesterol metabolites [20,22]. To the best of our knowledge, no direct study has been reported for Cyp27a1-driven cholesterol metabolites causing UPR.

**Figure 2:**
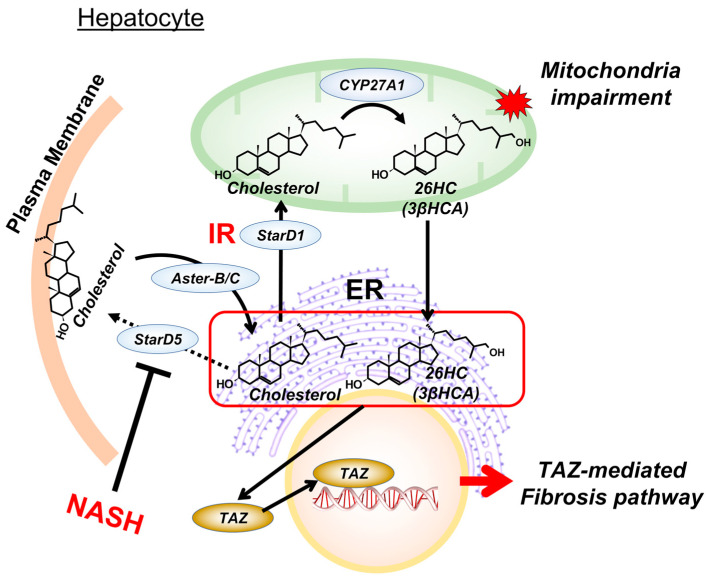
Role of cholesterol metabolites in endoplasmic reticulum function—extended two-hit hypothesis: Recent studies have provided evidence that increased levels of excess cholesterol in the endoplasmic reticulum (ER) are capable of activating fibrotic pathways. Supportive of these findings, Wang et al. demonstrated that Aster-B/C-protein-mediated cholesterol internalization from plasma membrane (PM) to the ER can activate TAZ-transcriptional fibrosis pathways [107]. Additionally, we have found StarD5, an ER stress-responsive transporter of cholesterol from the ER to the PM, appears to be downregulated in conditions of triglyceride excess [111,112]. This may also contribute to the accumulation of cholesterol in the ER. Under metabolic conditions of insulin resistance (IR), excess ER cholesterol can be transported to the mitochondria and converted into 26HC and 3βHCA, causing mitochondrial dysfunction. Both 26HC and 3βHCA can be rapidly transported to the ER. However, an inability to upregulate CYP7B1 under IR leads to the accumulation of 26HC/3βHCA in the ER. Therefore, it can be hypothesized that excess 26HC/3βHCA, together with cholesterol in the ER, might activate the TAZ-mediated fibrosis pathways under the conditions of chronic IR in NASH. This extended hypothesis introduces a novel concept of IR-induced hepatotoxicity and a mechanism of NAFLD disease progression. Abbreviations: 3βHCA, 3β-hydroxy-5-cholesten-(*25R*)26-oic acid; 26HC, (*25R*)26-hydroxycholesterol; StarD1, steroidogenic acute regulatory protein.

Perhaps ER cholesterol and Cyp27a1-driven cholesterol metabolites have important roles in the activation of fibrosis pathways (Figure 2). Wang et al. observed that the Aster B/C-protein-mediated internalization of plasma membrane cholesterol to the ER led to the TAZ-mediated transcriptional activation of fibrotic genes [107,108], hypothesizing excess cholesterol itself to activate fibrosis pathways. Meanwhile, we have recently found that StarD5, a steroidogenic regulatory protein-related lipid transfer (START) domain cholesterol-binding protein [109,110], plays a role in the assembly and secretion of both cholesterol and triglyceride from hepatocytes (unpublished data). In StarD5^−/−^ mice, cell triglycerides accumulate with the early development of IR while the mice are fed a low-fat, low-cholesterol rodent chow (unpublished data). For yet unclear reasons, we have also observed that liver StarD5 is downregulated in conditions with hepatic triglyceride excess [111,112]. Our preliminary findings of elevated 26HC/3βHCA in WD-fed StarD5^−/−^ mice support an increased intracellular trafficking of excess ER cholesterol to the mitochondria in NASH livers (unpublished data). With a disruption in 26HC/3βHCA metabolism due to impaired Cyp7b1 in the ER, excess 26HC/3βHCA in the ER can be hypothesized to activate TAZ-mediated fibrosis pathways (Figure 2). If this occurs, Cyp27a1-driven oxysterol accumulation represents a new player for developing fibrosis. It may then be possible to extend the current two-hit hypothesis to sequential events for what initiates the transition from fatty liver to NASH and fibrosis. This hypothesis is currently under investigation in our laboratories.

## 7. Targeting Mitochondrial Cholesterol Metabolites for NASH Intervention

The ability to safely store fats within the liver can be considered necessary for survival, as in the goose, which must store large amounts of fat to be able fly to the next migratory destination; this is not dissimilar to humans when food was not as abundant or when regular meals were a luxury. Additionally, not excluding fats as a direct cause of mitochondrial toxicity [113], new observations suggest a role of fats in NAFLD as the inducer of IR [23], which then leads to dysregulation in the cholesterol metabolism and the accumulation of the more likely toxic oxysterol metabolites. Therefore, following the discussion and the literature outlined in this manuscript, it is reasonable to hypothesize that either limiting the synthesis or facilitating the elimination of toxic mitochondrial cholesterol metabolites could improve hepatic inflammation in NAFLD. Table 2 lists the studies targeting mitochondria-driven cholesterol metabolites for the improvement of a NASH-related liver inflammatory condition. The liver-specific knockout of StarD1 in mice was shown to lessen steatohepatitis -riven HCC [82]. 

The beneficial effects of hepatocyte selective StarD1 deletion or downregulation have also been reported in many other animal models, including acetaminophen-mediated acute liver failure [120], Niemann–Pick Type C [121], and alcohol-induced steatohepatitis [122]. Acid ceramide appears to have a hepatic-StarD1-repressing effect [121]. Several small-molecule StarD1 inhibitors were also identified via structure-based design [123]. However, to the best of our knowledge, these molecules have never been tested for NASH interventions. Of importance, the global inhibition of StarD1 can be lethal, as the steroidogenic inhibition of StarD1 appears to disrupt vital steroid biosynthesis [124,125,126,127]. Perhaps the challenging part of testing these StarD1 inhibitors would be their selective delivery to the liver. 

Based on the observations from CTX patients [50,51], Cyp27a1^−/−^ mice [92,93], and goose fatty liver models [94], it is also reasonable to consider Cyp27a1 inhibition for reducing NASH inflammatory responses. Although no such studies have been reported for NASH intervention, Cyp27a1 inhibition is now being explored for the treatment of breast cancer [128,129] and toxic retinol accumulation in the eye [130]. Additionally, oral CDCA has successfully been used to lessen liver inflammation in patients with CYP7B1 deficiency [56,59,131]. CDCA has a CYP27A1 inhibitory effect. It should be mentioned that in humans, complete CYP27A1 inhibition increases cholesterol and 5α-cholestanol levels, causing CTX [132]. Therefore, CYP27A1 needs to be “partially” inhibited. Lam et al. have identified two FDA-approved drugs for hypertension (Felodipine and Nilvadipine) which have an estimated Cyp27a1 inhibitory effect of 50% [133]. The group reported that the administration of either Felodipine or Nilvadipine (1 mg/kg) for 7 days in C57Bl/6 mice did not elevate serum cholesterol levels. These two drugs are worth testing for their effectiveness in reducing NASH inflammatory responses.

Several studies have been reported in modulating oxysterol metabolic enzymes (i.e., Cyp7b1 and Sult2b1) with the purpose of improving NASH-related hepatic conditions. Bai et al. demonstrated that the adenovirus-mediated overexpression of Sult2b1 improved hepatic inflammation and stress responses in LDL^−/−^ mice and HFD-induced NAFL mouse models [114]. Similarly, Zhang et al. demonstrated that Sult2b1 overexpression in HFD-fed mice promoted liver regeneration after a 70% partial hepatectomy [115]. Although both studies did not measure hepatic oxysterol profiles, the increased sulfation of 26HC/3βHCA in the liver likely led to a reduction in their cytotoxicity. Additionally, the overexpression of Sult2b1 increases sulfation to endogenous 25HC, generating 25-Hydroxycholesterol-3-Sulfate (25HC3S) [114,134]. Ren and coworkers have demonstrated the implications of this sulfated oxysterol in many biological processes, including lipid biosynthesis, inflammatory responses, the promotion of cell survival, and recently, its potentially exciting role as an epigenetic regulator [134,135,136]. As listed in Table 2, the intraperitoneal injection of 25HC3S (25 mg/kg) twice per day for 6 weeks significantly improved liver function in HFD-fed mice with improved glucose tolerance [116]. Stabilization of the mitochondria was also reported for 25HC3S [137].

Similarly, the overexpression of Cyp7b1 has been found to lower fasting glucose levels and improve hepatic steatosis in *ob/ob* mice [138]. Currently, no small molecule that specifically upregulates Cyp7b1 is known. However, several agents (i.e., Psyllium husk, Ortlistat [118], and Ilexaponin A1 [119]) have been shown to be associated with the upregulation of Cyp7b1 in experimental NASH models (Table 2). Our group also demonstrated that the administration of berberine (50 mg/kg/day) in Western diet/high-fructose-diet-fed mice with induced NASH restored hepatic *Cyp7b1* mRNA [117]. The berberine-treated mice had significantly improved liver histology, NAS scores, and NASH-associated inflammation and stress responses. More recently, we have reported that dietary coffee preserved hepatic *Cyp7b1* and *Sult2b1* mRNA and protein expressions, thereby maintaining physiologic levels of 26HC/3βHCA in a WD-induced fatty liver mouse model [21]. The coffee-administered mice had markedly improved IR and early liver toxicity markers. Many other agents are reported to have a Cyp7b1-restoring effect in non-NASH animal models, such as ursodeoxycholic acid (UDCA) [139], Astragalus polysaccharides [140,141], Yinchenhao decoction [142], and ellagic acid [143]. These agents may be worth testing for their effectiveness in reducing early inflammatory responses in NAFLD.

Lastly, Farnesoid X receptor (FXR) agonists, such as obeticholic acid (OCA), appear to be a hopeful medication for NAFLD treatment because of their anti-inflammatory, insulin-sensitizing, anti-steatotic, and anti-fibrotic effects, which have been demonstrated in animal models [144]. However, the expressions of *Cyp27a1* and *Cyp7b1* are not regulated by FXR [52,69]; therefore, the beneficial effects of OCA are likely independent from the modulation of the mitochondrial cholesterol metabolisms. A recent study demonstrated no significant changes in the mRNA expression of *Cyp27a1* or *Cyp7b1* when mice were administrated an FXR agonist, GW4064 [145]. Meanwhile, liver X receptor (LXRα) activation has been attempted in many drug discovery studies for the treatment of dyslipidemia and atherosclerosis, but all of them have been failed due to LXR’s ability to stimulate de novo lipogenesis. Furthermore, the LXRα agonist possibly increases hepatic StarD1 expression, leads to mitochondrial 26HC/3βHCA accumulations, and worsens hepatic inflammation.

## 8. Conclusions and Remarks

The prevalence of NASH is rapidly increasing worldwide and has no approved pharmacological treatment to date. The literature reviewed herein supports the concept of a “two-hit” hypothesis, with the dysregulation of intracellular insulin signaling representing the “first hit” and the toxic accumulation of mitochondrial cholesterol metabolites representing a “second hit”, leading to initially toxic and subsequently inflammatory responses. This last observation is significant as very early transition from NAFL to NASH lacks any inflammatory cell infiltrate [20]. 

The restoration of insulin signaling as a treatment for diabetes is a beneficial NASH treatment [23]. However, the response to the agents is not always complete. As shown in recent clinical trials in patients with NAFLD [5,6,7,8,9,10,11], GLP-1 agonists induce weight loss and improve insulin sensitization yet are unable to lessen or reverse fibrosis. This inadequate response presumably occurs as an inability to fully correct cell insulin signaling, with continued production and dysregulation caused by cholesterol metabolites. Furthermore, appetite, which represents a living organism’s greatest survival drive, will undoubtedly be equally hard to fully control. As in the treatment of hypertension and hyperlipidemia, multiple pathways will likely need to be addressed to achieve a maximal response with the lowest toxicity profile. Several pharmaceutical agents are currently available for modifying hepatic 26HC/3βHCA, such as partial Cyp27a1 inhibitor [133], Cyp7b1 upregulators (i.e., berberine [117]) or 25HC3S [116,136]. The combined use of these agents with a GLP-1 agonist might have a dramatic effect on improving the hepatocyte inflammatory response in NASH, as does the addition of Ezetimibe (Zetia) to HMGR inhibitors [146,147]. Such tests in animal models and randomized controlled trials in patients with NASH or fibrosis are eagerly awaited.

It should be mentioned that the “two-hit hypothesis” can be extended to extrahepatic organs. Importantly, cardiovascular diseases are the leading cause of morbidity and mortality in patients with NAFLD. Endovascular atherosclerotic plaques contain a high level of 26HC [148]. The role of oxysterols and cholestenoic acids in macrophages may be closely related to the liver and may have an impact on macrophage cholesterol efflux [39] and the reverse cholesterol transport pathway [149]. It is possible that as in NAFLD, insufficient insulin signaling contributes to excess 26HC/3βHCA formation within cardiovascular tissue, leading to inflammation. 

The acidic pathway represents an “originating pathway” of bile acid synthesis whose essential purpose is to generate important cholesterol and lipid regulatory molecules (oxysterols and cholestenoic acids), with their subsequent further metabolism as a means of creating a nutritional advantage by adding bile acids to the intestinal milieu. However, the generation of excess amounts of potentially toxic molecules on the way to generating bile acids could be easily avoided by initially 7α-hydroxylating cholesterol via CYP7A1 (the neutral pathway); leaving the acidic pathway to its preeminent function. As was found with NAFLD, metabolic conditions exist that demonstrate how important it is to maintain physiological levels of mitochondria-driven cholesterol metabolites and their need to be constantly fluctuating to regulate changing hepatic lipid and cholesterol levels. Over the last century, our knowledge of cholesterol/bile acid chemistry and biology has been advanced extraordinarily [150]. We still need greater systematic and mechanistic understandings of these interesting biomolecules, which will most likely provide us with new avenues for therapeutic innovations in metabolic syndrome.

## Figures and Tables

**Table 1:** Mechanistic studies role of mitochondria-driven cholesterol metabolites in NASH development.

	Model	Main Findings in Terms of Role Oxysterols (Cholestenoic Acids) in NAFLD Pathogenesis	Pitfalls and Unresolved Question
Raselli et al. [64]	1. Biopsy-proven NASH	1. Elevated 24(R/S)HC and 7αHC but no changes in 25HC and 26HC levels in NASH livers compared with control livers without inflammation.	1. The data compared advanced NASH (NAS score 3–5; NASH fibrosis) and normal livers. No comparison is provided between steatosis and a normal liver.
	2. C57Bl/6; Ch25h^−/−^; Ebi2^−/−^; Cyp7b1^−/−^ mice fed with high-fat, high-cholesterol diet and high-fructose corn-syrup in their drinking water for 10–20 weeks.	2. Elevated 24(R/S)HC and 7αHC but no changes in 25HC and 26HC levels in NASH livers (C57Bl/6). No genotype-related differences were found in the development of NASH; no essential role of these gene expressions and/or 26HC in NASH pathogenesis.	2. The study focused on established NASH in which hepatic IR is no longer the key factor for oxysterol regulation.
Kakiyama et al. [20]	1. ♂ B6/129 mice fed with a Western diet for 2–6 weeks	1. Elevated liver 26HC/3βHCA and suppressed Cyp7b1 mRNA in early fatty liver without histologic inflammation. Correlated levels of 26HC/3βHCA to HOMA-IR sores and liver enzymes (ALT).	1. It remains unclear whether the elevated levels of 26HC/3βHCA themselves are direct causes of hepatotoxicity. Mechanistic study is missing.
	2. Streptozotocin-injected ♂ C57Bl/6 mice fed with LFD.	2. Plasma insulin level is directly correlated to hepatic Cyp7b1 mRNA expression.	2. Mechanism as to how insulin signal pathway regulates hepatic Cyp7b1 gene expression was not studied.
Na et al. [65]	♂ Catalase knockout mouse; C57Bl/6J fed with HFD for 11 weeks	Significantly reduced 3β,7α-diHCA secondary to Cyp7b1 mRNA downregulation in the livers of HFD-fed mice with higher serum ALT. The effect was more profound in Catalase knockout mice.	Histologic evaluation is missing and NAFLD stage is unclear; the presence of IR is unknown; the tissue 26HC/3βHCA level is unknown. The causative factor of liver injury cannot be proven solely by this study.
Evangelakos et al. [66]	♂ Cyp7b1^−/−^; C57Bl/6 littermates fed with a choline-deficient HFD and housed in a thermoneutral temperature for 8 months.	The thermoneutral housing of Cyp7b1^−/−^ mice promoted MAFLD more profoundly compared to the wild-type mice littermates. However, oxysterols did not correlate with the aggravation of MAFLD.	The model focused on established NASH, and oxysterol correlation with early disease progression is unknown. It is unclear whether choline-deficient HFD metabolically follows human metabolic disease.
Shoji et al. [67] Suga et al. [68]	♂ C57Bl/6J fed with choline-deficient, methionine-reduced high-fat diet for 3–21 days	When transitioning from NAFL to NASH, the hepatic desmosterol, 4βHC, secondary bile acid, etc., levels were significantly reduced. However, 26HC/3βHCA levels were unchanged.	Unclear whether choline-deficient HFD metabolically follows human metabolic disease.
Minowa et al. [22]	1. Biopsy-proven NASH patients	1. Elevated 26HC/3βHCA levels in NASH livers.	1. NASH patient cohort and the 26HC/3βHCA levels in early fatty liver remain unknown.
	2. ♂ Cyp7b1^−/−^ mice fed with WD and HCD for 4 weeks.	2. Elevated 24(S),25EC, 26HC, and 3βHCA in the WD-fed Cyp7b1^−/−^ mice with IR. Oxysterol sulfation and glucuronidation can also be impaired in early fatty liver with IR; contributing to the accumulation of 26HC/3βHCA.	2 and 3. There were no significant changes in the enzyme activities of oxidative phosphorylation with early NAFL, although these mRNAs were significantly downregulated. It remains unclear if oxysterol-associated mitochondrial dysfunction is the initial cause of hepatocyte injury.
	3. ♂ B6/129 mice fed with Western diet for 2–8 weeks	3. Accumulated 26HC/3βHCA in the liver mitochondria of NAFL mice with elevation of ALT. RNA-seq data showed that genes in mitochondria oxidative phosphorylation and thermogenesis are impaired.	

Abbreviations: ALT, alanine transferase; LFD, low-fat diet (normal rodent chow); HCD, high-cholesterol diet; HFD, high-fat diet. Ch25h^−/−^, microsomal cholesterol 25-hydroxylase knockout mouse; Ebi2^−/−^, Epstein–Barr-virus-induced G-protein-coupled receptor 2 knockout mouse; MAFLD, metabolic associated fatty liver disease.

**Table 2:** Studies modulating mitochondria-derived oxysterols for the improvement of the NAFLD condition.

Agent or Models	Animal Model and Treatment	Results and Oxysterol-Related Mechanism
Liver-specific StarD1 knockout mouse; (StarD1^Δhep^) mice [82]	Diethylnitorisamine (DEN; 25 mg/kg)-injected StarD1^Δhep^ (liver-specific StarD1 knockout mice) fed with a high-fat, high-cholesterol diet for 26 weeks.	StarD1^Δhep^ mice were less sensitive to DEN + HFHC-diet-induced HCC.
Ad-Sult2b1 [114]	LDL^−/−^ mice (♂/♀) and mice fed with a HFD for 10 weeks. Ad-Sult2b1 virus was injected (i.v., 1 × 10^8^) 6 days before sacrifice.	Improved NAFLD condition (i.e., ALT/AST↓ *; cholesterol/triglyceride↓; Steatosis (Histology)↓; hepatic inflammation and stress responses (mRNA)).
Ad-Sult2b1 [115]	Ad-Sult2b1 virus was injected (i.v., 1 × 10^8^) into C57Bl/6 mice, and the mice were fed a HFD for 8 weeks.	The Ad-Sult2b1-injected mice promoted liver regeneration after 70% partial hepatectomy.
25-Hydroxycholesterol 3-Sulfate [116]	C57BL/6J mice (♀) fed with a HFD for 16 weeks. As of 10 weeks after the HFD feeding was initiated, 25HC3S (25 mg/kg, i.p.) was injected twice a day for 6 weeks.	Improved NAFLD condition (i.e., ALT/AST↓; cholesterol/triglyceride↓; steatosis (histology)↓; improved glucose tolerance test).
Coffee [21]	B6/129 mice (♂) fed with 1% or 4% (*wt*/*wt*) regular coffee or decaffeinated coffee blended WD for two weeks.	The coffee-fed mice had significantly lower serum ALT and hepatic inflammatory mRNAs and improved HOMA-IR scores compared to the WD-only fed mice. These mice had reduced 26HC/3βHCA secondary to upregulated hepatic Sult2b1 and Cyp7b1 mRNAs.
Berberine [117]	B6/129 mice (♂/♀) were fed with a Western diet–high-fructose diet to up to 21 weeks. As of 12 weeks of age, Berberine (50 mg/kg/day) was administered via gavage.	Significantly improved histology, NAS scores, and NASH-associated inflammation and stress responses in BBR-treated mice. Higher hepatic Cyp7b1 mRNA in BBR-treated mice compared to the mice without treatment.
Psyllium husk (lipase inhibitor) [118]	C57Bl/6 (♂) mice fed a high-fat diet for 16 weeks. Psyllium husk (140 mg/kg) was administered via oral gavage three times/day.	Improved NAFLD condition (i.e., body weight↓; ALT/AST↓; cholesterol/triglyceride↓; NAS score↓; steatosis (histology)↓). Higher hepatic Cyp7b1 mRNA in the Psyllium-husk-treated mice compared to the mice without treatment.
Ortlistat (lipase inhibitor) [118]	C57Bl/6 (♂) mice fed a high-fat diet for 16 weeks. Ortlistat (20 mg/kg × 1 and 10 mg/kg × 2) was administered via oral gavage.	Improved NAFLD condition (i.e., body weight↓; ALT/AST↓; cholesterol/triglyceride↓; NAS score↓; steatosis (histology)↓). Higher hepatic Cyp7b1 mRNA in the Ortlistat-treated mice compared to the mice without treatment.
Ilexaponin A1 [119]	C57BL/6 (♂) mice fed a high-fat diet for 8 weeks. Ilexaponin A1 (120 mg/kg/day) was administered via oral gavage.	Improved NAFLD condition (i.e., ALT/AST↓; NAS score↓; steatosis (histology)↓; hepatic inflammatory genes↓) of the Ilexaponin-A1-administered mice. Higher hepatic Cyp7b1 mRNA in the Ilexaponin-A1-treated mice compared to the mice without treatment.

* Down-arrow (↓) indicates “decreased level”.

## Abbreviations

ApoE, apolipoprotein E; CA, cholic acid; CDCA, chenodeoxycholic acid; CTX, cerebrotendineous xanthomatosis; CYP7A1, cholesterol 7α-hydroxylase (Cyp7a1 denotes murine); CYP7B1, oxysterol 7α-hydroxylase (Cyp7b1 denotes murine); CYP8B1, sterol 12α-hydroxylase; CYP27A1, sterol 27-hydroxylase (Cyp27a1 denotes murine); C4, 7α-hydroxy-4-cholesten-3-one; diHC, dihydroxycholesterol; diCO, dihydroxy-4-cholest-3-one; ER, endoplasmic reticulum; GLP-1, glucagon-like peptide-1; HC, hydroxycholesterol; 3βHCA, 3β-hydroxy-5-cholest-(25R)26-oic acid; HCC, hepatocellular carcinoma; 25HC3S, 25-hydroxycholesterol-3β-sulfate; HFD, high-fat diet; HMG-CoA, hydroxy-methylglutaryl-CoA reductase; IMM, inner mitochondrial membrane; HNF4α, hepatocyte nuclear factor 4α; HSD3B7, 3β-hydroxy-Δ^5^-C27-steroid dehydrogenase/isomerase; IR, insulin resistance; LXR, liver X receptor; NAFL, nonalcoholic fatty liver; NAFLD, nonalcoholic fatty liver disease; NASH, nonalcoholic steatohepatitis; NAS score, NAFLD activity score; NPC, Niemann–Pick type C protein; SPG5, spastic paraplegia type 5; SREBP, sterol regulatory element-binding protein; OMM, outer mitochondrial membrane; StarD1, steroidogenic acute regulatory protein; Sult2b1, hydroxysteroid sulfotransferase 2b1; T2DM, type 2 diabetes mellitus; UDCA, ursodeoxycholic acid; Ugt, UDP-glucuronosyl transferase; UPR, unfolded protein response; WD, Western diet.

## References

[1] Ludwig J., Viggiano T.R., McGill D.B., Oh B.J. (1980). Nonalcoholic steatohepatitis: Mayo Clinic experiences with a hitherto unnamed disease. Mayo Clin. Proc..

[2] Younossi Z.M., Anstee Q.M., Marietti M., Hardy T., Henry L., Eslam M., George J., Bugianesi E. (2018). Global burden of NAFLD and NASH: Trends, predictions, risk factors and prevention. Nat. Rev. Gastroenterol. Hepatol..

[3] Riazi K., Azhari H., Charette J.H., Underwood E.F., King A.J., Afshar E.E., Swain M.G., Congly S.E., Kaplan G.G., Shaheen A.-A. (2022). The prevalence and incidence of NAFLD worldwide: A systematic review and meta-analysis. Lancet Gastroenterol. Hepatol..

[4] Younossi Z., Tacke F., Arrese M., Sharma B.C., Mostafa I., Bugianesi E., Wong V.W.-S., Yilmaz Y., George J., Fan J. (2019). Global Perspectives on Nonalcoholic Fatty Liver Disease and Nonalcoholic Steatohepatitis. Hepatology.

[5] Armstrong M.J., Gaunt P., Aithal G.P., Barton D., Hull D., Parker R., Hazlehurst J.M., Guo K., Abouda G., Aldersley A.M. (2016). Liraglutide safety and efficacy in patients with non-alcoholic steatohepatitis (LEAN): A multicentre, double-blind, randomised, placebo-controlled phase 2 study. Lancet.

[6] Zinman B., Wanner C., Lachin J.M., Fitchett D., Bluhmki E., Hantel S., Mattheus M., Devins T., Johansen O.E., Woerle H.J. (2015). Empagliflozin, Cardiovascular Outcomes, and Mortality in Type 2 Diabetes. N. Engl. J. Med..

[7] Marso S.P., Bain S.C., Consoli A., Eliaschewitz F.G., Jódar E., Leiter L.A., Lingvay I., Rosenstock J., Seufert J., Warren M.L. (2016). Semaglutide and Cardiovascular Outcomes in Patients with Type 2 Diabetes. N. Engl. J. Med..

[8] Marso S.P., Daniels G.H., Brown-Frandsen K., Kristensen P., Mann J.F.E., Nauck M.A., Nissen S.E., Pocock S., Poulter N.R., Ravn L.S. (2016). Liraglutide and Cardiovascular Outcomes in Type 2 Diabetes. N. Engl. J. Med..

[9] O’Neil P.M., Birkenfeld A.L., McGowan B., Mosenzon O., Pedersen S.D., Wharton S., Carson C.G., Jepsen C.H., Kabisch M., Wilding J.P.H. (2018). Efficacy and safety of semaglutide compared with liraglutide and placebo for weight loss in patients with obesity: A randomised, double-blind, placebo and active controlled, dose-ranging, phase 2 trial. Lancet.

[10] Kushner R.F., Calanna S., Davies M., Dicker D., Garvey W.T., Goldman B., Lingvay I., Thomsen M., Wadden T.A., Wharton S. (2020). Semaglutide 2.4 mg for the Treatment of Obesity: Key Elements of the STEP Trials 1 to 5. Obesity.

[11] Newsome P.N., Buchholtz K., Cusi K., Linder M., Okanoue T., Ratziu V., Sanyal A.J., Sejling A.-S., Harrison S.A. (2021). A Placebo-Controlled Trial of Subcutaneous Semaglutide in Nonalcoholic Steatohepatitis. N. Engl. J. Med..

[12] Powell E.E., Wong V.W.-S., Rinella M. (2021). Non-alcoholic fatty liver disease. Lancet.

[13] Ferrell J.M., Chiang J.Y.L. (2019). Understanding Bile Acid Signaling in Diabetes: From Pathophysiology to Therapeutic Targets. Diabetes Metab. J..

[14] Pandak W.M., Kakiyama G. (2019). The acidic pathway of bile acid synthesis: Not just an alternative pathway. Liver Res..

[15] Jia W., Wei M., Rajani C., Zheng X. (2021). Targeting the alternative bile acid synthetic pathway for metabolic diseases. Protein Cell.

[16] Horn C.L., Morales A.L., Savard C., Farrell G.C., Ioannou G.N. (2021). Role of Cholesterol-Associated Steatohepatitis in the Development of NASH. Hepatol. Commun..

[17] Kakiyama G., Marques D., Takei H., Nittono H., Erickson S., Fuchs M., Rodriguez-Agudo D., Gil G., Hylemon P.B., Zhou H. (2019). Mitochondrial oxysterol biosynthetic pathway gives evidence for CYP7B1 as controller of regulatory oxysterols. J. Steroid Biochem. Mol. Biol..

[18] Javitt N.B. (2002). Cholesterol, Hydroxycholesterols, and Bile Acids. Biochem. Biophys. Res. Commun..

[19] Griffiths W.J., Wang Y. (2022). Cholesterol metabolism: From lipidomics to immunology. J. Lipid Res..

[20] Kakiyama G., Marques D., Martin R., Takei H., Rodriguez-Agudo D., LaSalle S.A., Hashiguchi T., Liu X., Green R., Erickson S. (2020). Insulin resistance dysregulates CYP7B1 leading to oxysterol accumulation: A pathway for NAFL to NASH transition. J. Lipid Res..

[21] Kakiyama G., Minowa K., Rodriguez-Agudo D., Martin R., Takei H., Mitamura K., Ikegawa S., Mitsuyoshi S., Nittono H., Fuchs M. (2022). Coffee modulates insulin-HNF-4alpha-Cyp7b1 pathway and reduces oxysterol driven liver toxicity in a NAFLD mouse model. Am. J. Physiol. Gastrointest. Liver Physiol..

[22] Minowa K., Rodriguez-Agudo D., Suzuki M., Muto Y., Hirai S., Wang Y., Su L., Zhou H., Chen Q., Lesnefsky E.J. (2023). Insulin dysregulation drives mitochondrial cholesterol metabolite accumulation: Initiating hepatic toxicity in NAFLD. J. Lipid Res..

[23] Khan R.S., Bril F., Cusi K., Newsome P.N. (2019). Modulation of Insulin Resistance in Nonalcoholic Fatty Liver Disease. Hepatology.

[24] Watt M.J., Miotto P.M., De Nardo W., Montgomery M. (2019). The Liver as an Endocrine Organ—Linking NAFLD and Insulin Resistance. Endocr. Rev..

[25] Christenson L.K., McAllister J.M., Martin K.O., Javitt N.B., Osborne T.F., Strauss J.F. (1998). Oxysterol Regulation of Steroidogenic Acute Regulatory Protein Gene Expression. J. Biol. Chem..

[26] Dietschy J.M., Woollett L.A., Spady D.K. (1993). The Interaction of Dietary Cholesterol and Specific Fatty Acids in the Regulation of LDL Receptor Activity and Plasma LDL-Cholesterol Concentrations. Ann. New York Acad. Sci..

[27] Honda A., Miyazaki T., Iwamoto J., Hirayama T., Morishita Y., Monma T., Ueda H., Mizuno S., Sugiyama F., Takahashi S. (2020). Regulation of bile acid metabolism in mouse models with hydrophobic bile acid composition. J. Lipid Res..

[28] Li J., Dawson P.A. (2018). Animal models to study bile acid metabolism. Biochim. Biophys. Acta (BBA) Mol. Basis Dis..

[29] Elustondo P., Martin L.A., Karten B. (2017). Mitochondrial cholesterol import. Biochim. Biophys. Acta (BBA) Mol. Cell Biol. Lipids.

[30] Pandak W.M., Ren S., Marques D., Hall E., Redford K., Mallonee D., Bohdan P., Heuman D., Gil G., Hylemon P. (2002). Transport of Cholesterol into Mitochondria Is Rate-limiting for Bile Acid Synthesis via the Alternative Pathway in Primary Rat Hepatocytes. J. Biol. Chem..

[31] Ren S., Hylemon P.B., Marques D., Gurley E., Bodhan P., Hall E., Redford K., Gil G., Pandak W.M. (2004). Overexpression of cholesterol transporter StAR increasesin vivo rates of bile acid synthesis in the rat and mouse. Hepatology.

[32] Caron K.M., Soo S.-C., Wetsel W.C., Stocco D.M., Clark B.J., Parker K.L. (1997). Targeted disruption of the mouse gene encoding steroidogenic acute regulatory protein provides insights into congenital lipoid adrenal hyperplasia. Proc. Natl. Acad. Sci. USA.

[33] Ren S., Hylemon P., Marques D., Hall E., Redford K., Gil G., Pandak W.M. (2004). Effect of increasing the expression of cholesterol transporters (StAR, MLN64, and SCP-2) on bile acid synthesis. J. Lipid Res..

[34] Balboa E., Castro J., Pinochet M.-J., Cancino G.I., Matías N., Sáez P.J., Martínez A., Álvarez A.R., Garcia-Ruiz C., Fernández-Checa J.C. (2017). MLN64 induces mitochondrial dysfunction associated with increased mitochondrial cholesterol content. Redox Biol..

[35] Fakheri R.J., Javitt N.B. (2012). 27-Hydroxycholesterol, does it exist? On the nomenclature and stereochemistry of 26-hydroxylated sterols. Steroids.

[36] Axelson M., Mörk B., Sjövall J. (1988). Occurrence of 3 beta-hydroxy-5-cholestenoic acid, 3 beta,7 alpha-dihydroxy-5-cholestenoic acid, and 7 alpha-hydroxy-3-oxo-4-cholestenoic acid as normal constituents in human blood. J. Lipid Res..

[37] Axelson M., Mörk B., Aly A., Wisén O., Sjövall J. (1989). Concentrations of cholestenoic acids in plasma from patients with liver disease. J. Lipid Res..

[38] Axelson M., Mörk B., Aly A., Walldius G., Sjövall J. (1989). Concentrations of cholestenoic acids in plasma from patients with reduced intestinal reabsorption of bile acids. J. Lipid Res..

[39] Taylor J.M., Borthwick F., Bartholomew C., Graham A. (2010). Overexpression of steroidogenic acute regulatory protein increases macrophage cholesterol efflux to apolipoprotein AI. Cardiovasc. Res..

[40] Song C., Liao S. (2000). Cholestenoic Acid Is a Naturally Occurring Ligand for Liver X Receptor α**This work was supported by NIH grants. Endocrinology.

[41] Fu X., Menke J.G., Chen Y., Zhou G., MacNaul K.L., Wright S.D., Sparrow C.P., Lund E.G. (2001). 27-Hydroxycholesterol Is an Endogenous Ligand for Liver X Receptor in Cholesterol-loaded Cells. J. Biol. Chem..

[42] Saito H., Tachiura W., Nishimura M., Shimizu M., Sato R., Yamauchi Y. (2023). Hydroxylation site–specific and production-dependent effects of endogenous oxysterols on cholesterol homeostasis: Implications for SREBP-2 and LXR. J. Biol. Chem..

[43] Heverin M., Ali Z., Olin M., Tillander V., Joibari M.M., Makoveichuk E., Leitersdorf E., Warner M., Olivercrona G., Gustafsson J. (2017). On the regulatory importance of 27-hydroxycholesterol in mouse liver. J. Steroid Biochem. Mol. Biol..

[44] Javitt N.B., Kok E., Cohen B., Burstein S. (1982). Cerebrotendinous xanthomatosis: Reduced serum 26-hydroxycholesterol. J. Lipid Res..

[45] Björkhem I., Hansson M. (2010). Cerebrotendinous xanthomatosis: An inborn error in bile acid synthesis with defined mutations but still a challenge. Biochem. Biophys. Res. Commun..

[46] Shen C.-H., Wang Z.-X. (2018). Liver transplantation due to cerebrotendinous xanthomatosis end-stage liver disease. World J. Pediatr..

[47] Pietrobattista A., Spada M., Candusso M., Boenzi S., Dionisi-Vici C., Francalanci P., Morrone A., Ferri L., Indolfi G., Agolini E. (2022). Liver transplantation in an infant with cerebrotendinous xanthomatosis, cholestasis, and rapid evolution of liver failure. Pediatr. Transplant..

[48] Lipiński P., Klaudel-Dreszler M., Ciara E., Jurkiewicz D., Płoski R., Cielecka-Kuszyk J., Socha P., Jankowska I. (2020). Sterol 27-Hydroxylase Deficiency as a Cause of Neonatal Cholestasis: Report of 2 Cases and Review of the Literature. Front. Pediatr..

[49] Gong J.-Y., Setchell K.D., Zhao J., Zhang W., Wolfe B., Lu Y., Lackner K., Knisely A., Wang N.-L., Hao C.-Z. (2017). Severe Neonatal Cholestasis in Cerebrotendinous Xanthomatosis: Genetics, Immunostaining, Mass Spectrometry. J. Craniofacial Surg..

[50] Heubi J.E., Setchell K.D.R., Bove K.E. (2007). Inborn Errors of Bile Acid Metabolism. Semin. Liver Dis..

[51] Duell P.B., Salen G., Eichler F.S., DeBarber A.E., Connor S.L., Casaday L., Jayadev S., Kisanuki Y., Lekprasert P., Malloy M.J. (2018). Diagnosis, treatment, and clinical outcomes in 43 cases with cerebrotendinous xanthomatosis. J. Clin. Lipidol..

[52] Wu Z., Martin K., Javitt N., Chiang J. (1999). Structure and functions of human oxysterol 7α-hydroxylase cDNAs and gene CYP7B1. J. Lipid Res..

[53] Stiles A.R., McDonald J.G., Bauman D.R., Russell D.W. (2009). CYP7B1: One Cytochrome P450, Two Human Genetic Diseases, and Multiple Physiological Functions. J. Biol. Chem..

[54] Ayaki Y., Kok E., Javitt N.B. (1989). Cholic Acid Synthesis from 26-Hydroxycholesterol and 3-Hydroxy-5-cholestenoic Acid in the Rabbit. J. Biol. Chem..

[55] Setchell K.D.R., Schwarz M., O’Connell N.C., Lund E.G., Davis D.L., Lathe R., Thompson H.R., Tyson R.W., Sokol R.J., Russell D.W. (1998). Identification of a new inborn error in bile acid synthesis: Mutation of the oxysterol 7alpha-hydroxylase gene causes severe neonatal liver disease. J. Clin. Investig..

[56] Dai D., Mills P.B., Footitt E., Gissen P., McClean P., Stahlschmidt J., Coupry I., Lavie J., Mochel F., Goizet C. (2014). Liver disease in infancy caused by oxysterol 7α-hydroxylase deficiency: Successful treatment with chenodeoxycholic acid. J. Inherit. Metab. Dis..

[57] Mizuochi T., Kimura A., Suzuki M., Ueki I., Takei H., Nittono H., Kakiuchi T., Shigeta T., Sakamoto S., Fukuda A. (2011). Successful heterozygous living donor liver transplantation for an oxysterol 7α-hydroxylase deficiency in a Japanese patient. Liver Transplant..

[58] Kobayashi M., Koike M., Sakiyama M., Okuda S., Okuda M., Tanaka T., Unno A., Nittono H., Takei H., Murai T. (2000). 3beta-Hydroxy-Delta5-C27-steroid dehydrogenase/isomerase deficiency in a 23-year-old woman. Pediatr. Int..

[59] Kimura A., Mizuochi T., Takei H., Ohtake A., Mori J., Shinoda K., Hashimoto T., Kasahara M., Togawa T., Murai T. (2021). Bile Acid Synthesis Disorders in Japan: Long-Term Outcome and Chenodeoxycholic Acid Treatment. Dig. Dis. Sci..

[60] Li-Hawkins J., Lund E.G., Turley S.D., Russell D.W. (2000). Disruption of the Oxysterol 7α-Hydroxylase Gene in Mice. J. Biol. Chem..

[61] Minowa K., Rodriguez-Agudo D., Suzuki M., Hirai S., Muto Y., Su L., Mitamura K., Ikegawa S., Heuman D.M., Zhou H. (2022). Modulation of Mitochondrial Cholesterol Metabolites in Cyp7b1 Deficiency: A New Insight into a Mechanism of Initiating Nash. Hepatology.

[62] Houben T., Bitorina A.V., Oligschlaeger Y., Jeurissen M.L., Rensen S., Köhler S.E., Westerterp M., Lütjohann D., Theys J., Romano A. (2020). Sex-opposed inflammatory effects of 27-hydroxycholesterol are mediated via differences in estrogen signaling. J. Pathol..

[63] Farrell G., Schattenberg J.M., Leclercq I., Yeh M.M., Goldin R., Teoh N., Schuppan D. (2018). Mouse Models of Nonalcoholic Steatohepatitis: Toward Optimization of Their Relevance to Human Nonalcoholic Steatohepatitis. Hepatology.

[64] Raselli T., Hearn T., Wyss A., Atrott K., Peter A., Frey-Wagner I., Spalinger M.R., Maggio E.M., Sailer A.W., Schmitt J. (2019). Elevated oxysterol levels in human and mouse livers reflect nonalcoholic steatohepatitis. J. Lipid Res..

[65] Na J., Choi S.A., Khan A., Huh J.Y., Piao L., Hwang I., Ha H., Park Y.H. (2019). Integrative Omics Reveals Metabolic and Transcriptomic Alteration of Nonalcoholic Fatty Liver Disease in Catalase Knockout Mice. Biomol. Ther..

[66] Evangelakos I., Schwinge D., Worthmann A., John C., Roeder N., Pertzborn P., Behrens J., Schramm C., Scheja L., Heeren J. (2021). Oxysterol 7-α Hydroxylase (CYP7B1) Attenuates Metabolic-Associated Fatty Liver Disease in Mice at Thermoneutrality. Cells.

[67] Shoji S., Maekawa M., Ogura J., Sato T., Mano N. (2022). Identification cholesterol metabolites altered before the onset of nonalcoholic steatohepatitis by targeted metabolomics. Biochim. Biophys. Acta (BBA) Mol. Cell Biol. Lipids.

[68] Suga T., Yamaguchi H., Ogura J., Shoji S., Maekawa M., Mano N. (2019). Altered bile acid composition and disposition in a mouse model of non-alcoholic steatohepatitis. Toxicol. Appl. Pharmacol..

[69] Wu Z., Chiang J.Y. (2001). Transcriptional regulation of human oxysterol 7α-hydroxylase gene (CYP7B1) by Sp1. Gene.

[70] Inoue Y., Yu A.-M., Yim S.H., Ma X., Krausz K.W., Inoue J., Xiang C.C., Brownstein M.J., Eggertsen G., Björkhem I. (2006). Regulation of bile acid biosynthesis by hepatocyte nuclear factor 4α. J. Lipid Res..

[71] Gupta R.K., Kaestner K.H. (2004). HNF-4α: From MODY to late-onset type 2 diabetes. Trends Mol. Med..

[72] Pass G.J., Becker W., Kluge R., Linnartz K., Plum L., Giesen K., Joost H.-G., Anderson J.J., Rao S.P., Rowe B. (2002). Effect of Hyperinsulinemia and Type 2 Diabetes-Like Hyperglycemia on Expression of Hepatic Cytochrome P450 and Glutathione*S*-Transferase Isoforms in a New Zealand Obese-Derived Mouse Backcross Population. Experiment.

[73] Nojima K., Sugimoto K., Ueda H., Babaya N., Ikegami H., Rakugi H. (2013). Analysis of hepatic gene expression profile in a spontaneous mouse model of type 2 diabetes under a high sucrose diet. Endocr. J..

[74] Biddinger S.B., Haas J.T., Yu B.B., Bezy O., Jing E., Zhang W., Unterman T.G., Carey M.C., Kahn C.R. (2008). Hepatic insulin resistance directly promotes formation of cholesterol gallstones. Nat. Med..

[75] Tang W., Pettersson H., Norlin M. (2008). Involvement of the PI3K/Akt pathway in estrogen-mediated regulation of human CYP7B1: Identification of CYP7B1 as a novel target for PI3K/Akt and MAPK signalling. J. Steroid Biochem. Mol. Biol..

[76] Bi Y., Shi X., Zhu J., Guan X., Garbacz W.G., Huang Y., Gao L., Yan J., Xu M., Ren S. (2018). Regulation of Cholesterol Sulfotransferase SULT2B1b by Hepatocyte Nuclear Factor 4*α* Constitutes a Negative Feedback Control of Hepatic Gluconeogenesis. Mol. Cell Biol..

[77] Coll O., Colell A., García-Ruiz C., Kaplowitz N., Fernández-Checa J.C. (2003). Sensitivity of the 2-oxoglutarate carrier to alcohol intake contributes to mitochondrial glutathione depletion. Hepatology.

[78] Van Meer G., Voelker D.R., Feigenson G.W. (2008). Membrane lipids: Where they are and how they behave. Nat. Rev. Mol. Cell Biol..

[79] Caballero F., Fernández A., De Lacy A.M., Fernández-Checa J.C., Caballería J., García-Ruiz C. (2009). Enhanced free cholesterol, SREBP-2 and StAR expression in human NASH. J. Hepatol..

[80] Chen W., Chiang J.Y. (2003). Regulation of human sterol 27-hydroxylase gene (CYP27A1) by bile acids and hepatocyte nuclear factor 4α (HNF4α). Gene.

[81] Hall E., Ren S., Hylemon P., Rodriguez-Agudo D., Redford K., Marques D., Kang D., Gil G., Pandak W. (2005). Detection of the steroidogenic acute regulatory protein, StAR, in human liver cells. Biochim. Biophys. Acta (BBA) Mol. Cell Biol. Lipids.

[82] de la Rosa L.C., Garcia-Ruiz C., Vallejo C., Baulies A., Nuñez S., Monte M.J., Marin J.J., Baila-Rueda L., Cenarro A., Civeira F. (2021). STARD1 promotes NASH-driven HCC by sustaining the generation of bile acids through the alternative mitochondrial pathway. J. Hepatol..

[83] Marí M., Caballero F., Colell A., Morales A., Caballeria J., Fernandez A., Enrich C., Fernandez-Checa J.C., García-Ruiz C. (2006). Mitochondrial free cholesterol loading sensitizes to TNF- and Fas-mediated steatohepatitis. Cell Metab..

[84] Tichauer M.J.E., Morales L.A.A.G., Amigo L., Galdames L., Klein V.S., Quiñones C.F.N., Ferrada C., Alvarez A., Rio M.-C., Miquel J.F. (2007). Overexpression of the cholesterol-binding protein MLN64 induces liver damage in the mouse. World J. Gastroenterol..

[85] Colell A., García-Ruiz C., Lluis J.M., Coll O., Mari M., Fernández-Checa J.C. (2003). Cholesterol Impairs the Adenine Nucleotide Translocator-mediated Mitochondrial Permeability Transition through Altered Membrane Fluidity. J. Biol. Chem..

[86] Wu M.K., Cohen D.E. (2005). Altered hepatic cholesterol metabolism compensates for disruption of phosphatidylcholine transfer protein in mice. Am. J. Physiol. Liver Physiol..

[87] Echegoyen S., Oliva E.B., Sepulveda J., Díaz-Zagoya J.C., Espinosa-García M.T., Pardo J.P., Martínez F. (1993). Cholesterol increase in mitochondria: Its effect on inner-membrane functions, submitochondrial localization and ultrastructural morphology. Biochem. J..

[88] Garcia-Ruiz C., Mari M., Colell A., Morales A., Caballero F., Montero J., Terrones O., Basañez G., Fernández-Checa J.C. (2009). Mitochondrial cholesterol in health and disease. Histol Histopathol..

[89] Ha S.-D., Park S., Han C.Y., Nguyen M.L., Kim S.O. (2012). Cellular Adaptation to Anthrax Lethal Toxin-Induced Mitochondrial Cholesterol Enrichment, Hyperpolarization, and Reactive Oxygen Species Generation through Downregulating MLN64 in Macrophages. Mol. Cell Biol..

[90] Marí M., Morales A., Colell A., García-Ruiz C., Fernández-Checa J.C. (2009). Mitochondrial Glutathione, a Key Survival Antioxidant. Antioxid. Redox Signal..

[91] Solsona-Vilarrasa E., Fucho R., Torres S., Nuñez S., Nuño-Lámbarri N., Enrich C., García-Ruiz C., Fernández-Checa J.C. (2019). Cholesterol enrichment in liver mitochondria impairs oxidative phosphorylation and disrupts the assembly of respiratory supercomplexes. Redox Biol..

[92] Zurkinden L., Mansour Y.T., Rohrbach B., Vogt B., Mistry H.D., Escher G. (2016). Hepatic caveolin-1 is enhanced in *Cyp27a1/ApoE* double knockout mice. FEBS Open Bio.

[93] Zurkinden L., Sviridov D., Vogt B., Escher G. (2018). Sterol 27-hydroxylase gene dosage and the antiatherosclerotic effect of Rifampicin in mice. Biosci. Rep..

[94] Zhao M., Xing Y., Liu L., Fan X., Liu L., Geng T., Gong D. (2020). GC-TOF-MS-Based Metabolomics Analyses of Liver and Intestinal Contents in the Overfed vs. Normally-Fed Geese. Animals.

[95] Musso G., Gambino R., Cassader M. (2013). Cholesterol metabolism and the pathogenesis of non-alcoholic steatohepatitis. Prog. Lipid Res..

[96] Serviddio G., Blonda M., Bellanti F., Villani R., Iuliano L., Vendemiale G. (2013). Oxysterols and redox signaling in the pathogenesis of non-alcoholic fatty liver disease. Free. Radic. Res..

[97] Bellanti F., Villani R., Tamborra R., Blonda M., Iannelli G., di Bello G., Facciorusso A., Poli G., Iuliano L., Avolio C. (2017). Synergistic interaction of fatty acids and oxysterols impairs mitochondrial function and limits liver adaptation during nafld progression. Redox Biol..

[98] Bellanti F., Mitarotonda D., Tamborra R., Blonda M., Iannelli G., Petrella A., Sanginario V., Iuliano L., Vendemiale G., Serviddio G. (2014). Oxysterols induce mitochondrial impairment and hepatocellular toxicity in non-alcoholic fatty liver disease. Free. Radic. Biol. Med..

[99] Zhang X., Wu X., Hu Q., Wu J., Wang G., Hong Z., Ren J. (2019). Mitochondrial DNA in liver inflammation and oxidative stress. Life Sci..

[100] Yu Y., Liu Y., An W., Song J., Zhang Y., Zhao X. (2019). STING-mediated inflammation in Kupffer cells contributes to progression of nonalcoholic steatohepatitis. J. Clin. Investig..

[101] Umetani M., Ghosh P., Ishikawa T., Umetani J., Ahmed M., Mineo C., Shaul P.W. (2014). The Cholesterol Metabolite 27-Hydroxycholesterol Promotes Atherosclerosis via Proinflammatory Processes Mediated by Estrogen Receptor Alpha. Cell Metab..

[102] Ajoolabady A., Kaplowitz N., Lebeaupin C., Kroemer G., Kaufman R.J., Malhi H., Ren J. (2023). Endoplasmic reticulum stress in liver diseases. Hepatology.

[103] Lebeaupin C., Vallée D., Hazari Y., Hetz C., Chevet E., Bailly-Maitre B. (2018). Endoplasmic reticulum stress signalling and the pathogenesis of non-alcoholic fatty liver disease. J. Hepatol..

[104] Gentile C.L., Frye M., Pagliassotti M.J. (2011). Endoplasmic Reticulum Stress and the Unfolded Protein Response in Nonalcoholic Fatty Liver Disease. Antioxid. Redox Signal..

[105] Fu S., Yang L., Li P., Hofmann O., Dicker L., Hide W., Lin X., Watkins S.M., Ivanov A.R., Hotamisligil G.S. (2011). Aberrant lipid metabolism disrupts calcium homeostasis causing liver endoplasmic reticulum stress in obesity. Nature.

[106] Park S.W., Zhou Y., Lee J., Lee J., Ozcan U. (2010). Sarco(endo)plasmic reticulum Ca^2+^ -ATPase 2b is a major regulator of endoplasmic reticulum stress and glucose homeostasis in obesity. Proc. Natl. Acad. Sci. USA.

[107] Wang X., Cai B., Yang X., Sonubi O.O., Zheng Z., Ramakrishnan R., Shi H., Valenti L., Pajvani U.B., Sandhu J. (2020). Cholesterol Stabilizes TAZ in Hepatocytes to Promote Experimental Non-alcoholic Steatohepatitis. Cell Metab..

[108] Wang X., Zheng Z., Caviglia J.M., Corey K.E., Herfel T.M., Cai B., Masia R., Chung R.T., Lefkowitch J.H., Schwabe R.F. (2016). Hepatocyte TAZ/WWTR1 Promotes Inflammation and Fibrosis in Nonalcoholic Steatohepatitis. Cell Metab..

[109] Rodriguez-Agudo D., Ren S., Hylemon P.B., Redford K., Natarajan R., Del Castillo A., Gil G., Pandak W.M. (2005). Human StarD5, a cytosolic StAR-related lipid binding protein. J. Lipid Res..

[110] Rodriguez-Agudo D., Ren S., Hylemon P.B., Montañez R., Redford K., Natarajan R., Medina M.A., Gil G., Pandak W.M. (2006). Localization of StarD5 cholesterol binding protein. J. Lipid Res..

[111] Rodriguez-Agudo D., Malacrida L., Kakiyama G., Sparrer T., Fortes C., Maceyka M., Subler M.A., Windle J.J., Gratton E., Pandak W.M. (2019). StarD5: An ER stress protein regulates plasma membrane and intracellular cholesterol homeostasis. J. Lipid Res..

[112] Rodriguez-Agudo D., Kakiyama G., Pandak Jr W.M. (2022). StarD5, Maintains Cholesterol and Lipid Homeostasis through its ability to Transport Cholesterol to the Pm and Increase Vldl Secretion: Protecting against Transition from Nafl to Nash. Hepatology.

[113] Fromenty B., Roden M. (2022). Mitochondrial alterations in fatty liver diseases. J. Hepatol..

[114] Bai Q., Zhang X., Xu L., Kakiyama G., Heuman D., Sanyal A., Pandak W.M., Yin L., Xie W., Ren S. (2012). Oxysterol sulfation by cytosolic sulfotransferase suppresses liver X receptor/sterol regulatory element binding protein–1c signaling pathway and reduces serum and hepatic lipids in mouse models of nonalcoholic fatty liver disease. Metabolism.

[115] Zhang X., Xu Y., Bai Q., Li X., Han J., Hou Y., Ji Y., Zhang Z. (2020). Inhibition of LXR signaling by SULT2B1b promotes liver regeneration after partial hepatectomy in mouse models of nonalcoholic fatty liver disease. Am. J. Physiol. Liver Physiol..

[116] Xu L., Kim J.K., Bai Q., Zhang X., Kakiyama G., Min H.-K., Sanyal A.J., Pandak W.M., Ren S. (2012). 5-Cholesten-3β,25-Diol 3-Sulfate Decreases Lipid Accumulation in Diet-Induced Nonalcoholic Fatty Liver Disease Mouse Model. Mol. Pharmacol..

[117] Wang Y., Tai Y.-L., Zhao D., Zhang Y., Yan J., Kakiyama G., Wang X., Gurley E.C., Liu J., Liu J. (2021). Berberine Prevents Disease Progression of Nonalcoholic Steatohepatitis through Modulating Multiple Pathways. Cells.

[118] Deng Z., Meng C., Huang H., Song S., Fu L., Fu Z. (2022). The different effects of psyllium husk and orlistat on weight control, the amelioration of hypercholesterolemia and non-alcohol fatty liver disease in obese mice induced by a high-fat diet. Food Funct..

[119] Zhao W.-W., Xiao M., Wu X., Li X.-W., Li X.-X., Zhao T., Yu L., Chen X.-Q. (2021). Ilexsaponin A1 Ameliorates Diet-Induced Nonalcoholic Fatty Liver Disease by Regulating Bile Acid Metabolism in Mice. Front. Pharmacol..

[120] Torres S., Baulies A., Insausti-Urkia N., Alarcón-Vila C., Fucho R., Solsona-Vilarrasa E., Núñez S.T., Robles D., Ribas V., Wakefield L. (2019). Endoplasmic Reticulum Stress-Induced Upregulation of STARD1 Promotes Acetaminophen-Induced Acute Liver Failure. Gastroenterology.

[121] Torres S., Solsona-Vilarrasa E., Nuñez S., Matías N., Insausti-Urkia N., Castro F., Casasempere M., Fabriás G., Casas J., Enrich C. (2021). Acid ceramidase improves mitochondrial function and oxidative stress in Niemann-Pick type C disease by repressing STARD1 expression and mitochondrial cholesterol accumulation. Redox Biol..

[122] Fernandez A., Matias N., Fucho R., Ribas V., Von Montfort C., Nuño N., Baulies A., Martinez L., Tarrats N., Mari M. (2013). ASMase is required for chronic alcohol induced hepatic endoplasmic reticulum stress and mitochondrial cholesterol loading. J. Hepatol..

[123] Akula N., Midzak A., Lecanu L., Papadopoulos V. (2012). Identification of small-molecule inhibitors of the steroidogenic acute regulatory protein (STARD1) by structure-based design. Bioorganic Med. Chem. Lett..

[124] Bhangoo A., Anhalt H., Ten S., King S.R. (2006). Phenotypic variations in lipoid congenital adrenal hyperplasia. Pediatr. Endocrinol. Rev..

[125] Baker B.Y., Lin L., Kim C.J., Raza J., Smith C.P., Miller W.L., Achermann J.C. (2006). Nonclassic Congenital Lipoid Adrenal Hyperplasia: A New Disorder of the Steroidogenic Acute Regulatory Protein with Very Late Presentation and Normal Male Genitalia. J. Clin. Endocrinol. Metab..

[126] Metherell L.A., Naville D., Halaby G., Begeot M., Huebner A., Nürnberg G., Nürnberg P., Green J., Tomlinson J., Krone N. (2009). Nonclassic Lipoid Congenital Adrenal Hyperplasia Masquerading as Familial Glucocorticoid Deficiency. J. Clin. Endocrinol. Metab..

[127] Khoury K., Barbar E., AinMelk Y., Ouellet A., Lavigne P., Lehoux J.-G. (2016). Thirty-Eight-Year Follow-Up of Two Sibling Lipoid Congenital Adrenal Hyperplasia Patients Due to Homozygous Steroidogenic Acute Regulatory (STARD1) Protein Mutation. Molecular Structure and Modeling of the STARD1 L275P Mutation. Front. Neurosci..

[128] Going C.C., Alexandrova L., Lau K., Yeh C.Y., Feldman D., Pitteri S.J. (2017). Vitamin D supplementation decreases serum 27-hydroxycholesterol in a pilot breast cancer trial. Breast Cancer Res. Treat..

[129] Mast N., Lin J.B., Pikuleva I.A. (2015). Marketed Drugs Can Inhibit Cytochrome P450 27A1, a Potential New Target for Breast Cancer Adjuvant Therapy. Mol. Pharmacol..

[130] Mast N., Reem R., Bederman I., Huang S., DiPatre P.L., Björkhem I., Pikuleva I. (2011). Cholestenoic Acid Is an Important Elimination Product of Cholesterol in the Retina: Comparison of Retinal Cholesterol Metabolism with That in the Brain. Investig. Opthalmol. Vis. Sci..

[131] Tang Y.-P., Gong J.-Y., Setchell K.D.R., Zhang W., Zhao J., Wang J.-S. (2021). Successful treatment of infantile oxysterol 7α-hydroxylase deficiency with oral chenodeoxycholic acid. BMC Gastroenterol..

[132] Björkhem I. (2013). Cerebrotendinous xanthomatosis. Curr. Opin. Infect. Dis..

[133] Lam M., Mast N., Pikuleva I.A. (2018). Drugs and Scaffold That Inhibit Cytochrome P450 27A1 In Vitro and In Vivo. Mol. Pharmacol..

[134] Bai Q., Xu L., Kakiyama G., Runge-Morris M.A., Hylemon P.B., Yin L., Pandak W.M., Ren S. (2011). Sulfation of 25-hydroxycholesterol by SULT2B1b decreases cellular lipids via the LXR/SREBP-1c signaling pathway in human aortic endothelial cells. Atherosclerosis.

[135] Wang Y., Li X., Ren S. (2020). Cholesterol Metabolites 25-Hydroxycholesterol and 25-Hydroxycholesterol 3-Sulfate Are Potent Paired Regulators: From Discovery to Clinical Usage. Metabolites.

[136] Wang Y., Lin W., Brown J.E., Chen L., Pandak W.M., Hylemon P.B., Ren S. (2021). 25-Hydroxycholesterol 3-sulfate is an endogenous ligand of DNA methyltransferases in hepatocytes. J. Lipid Res..

[137] Wang Y., Pandak W.M., Lesnefsky E.J., Hylemon P.B., Ren S. (2021). 25-Hydroxycholesterol 3-Sulfate Recovers Acetaminophen Induced Acute Liver Injury via Stabilizing Mitochondria in Mouse Models. Cells.

[138] Fu S., Fan J., Blanco J., Giménez-Cassina A., Danial N.N., Watkins S.M., Hotamisligil G.S. (2012). Polysome Profiling in Liver Identifies Dynamic Regulation of Endoplasmic Reticulum Translatome by Obesity and Fasting. PLoS Genet..

[139] Ma H., Sales V.M., Wolf A.R., Subramanian S., Matthews T.J., Chen M., Sharma A., Gall W., Kulik W., Cohen D.E. (2017). Attenuated Effects of Bile Acids on Glucose Metabolism and Insulin Sensitivity in a Male Mouse Model of Prenatal Undernutrition. Endocrinology.

[140] Zhuang Q., Ye X., Shen S., Cheng J., Shi Y., Wu S., Xia J., Ning M., Dong Z., Wan X. (2021). Astragalus Polysaccharides Ameliorate Diet-Induced Gallstone Formation by Modulating Synthesis of Bile Acids and the Gut Microbiota. Front. Pharmacol..

[141] Sun L., Pang Y., Wang X., Wu Q., Liu H., Liu B., Liu G., Ye M., Kong W., Jiang C. (2019). Ablation of gut microbiota alleviates obesity-induced hepatic steatosis and glucose intolerance by modulating bile acid metabolism in hamsters. Acta Pharm. Sin. B.

[142] Meng Y., Meng K., Zhao X., Li N., Gao Q., Wu S., Cui Y. (2018). Protective Effects of Yinchenhao Decoction on Cholesterol Gallstone in Mice Fed a Lithogenic Diet by Regulating LXR, CYP7A1, CYP7B1, and HMGCR Pathways. Evid. Based Complement Altern. Med..

[143] Duan J., Pan J., Sun M., Fang Y. (2022). Comparative multiomics study of the effects of Ellagic acid on the gut environment in young and adult mice. Food Res. Int..

[144] Steinman J.B., Salomao M.A., Pajvani U.B. (2021). Zonation in NASH—A key paradigm for understanding pathophysiology and clinical outcomes. Liver Int..

[145] Rizzolo D., Kong B., Taylor R.E., Brinker A., Goedken M., Buckley B., Guo G.L. (2021). Bile acid homeostasis in female mice deficient in Cyp7a1 and Cyp27a1. Acta Pharm. Sin. B.

[146] Takeshita Y., Takamura T., Honda M., Kita Y., Zen Y., Kato K.-I., Misu H., Ota T., Nakamura M., Yamada K. (2014). The effects of ezetimibe on non-alcoholic fatty liver disease and glucose metabolism: A randomised controlled trial. Diabetologia.

[147] Cho Y., Rhee H., Kim Y.-E., Lee M., Lee B.-W., Kang E.S., Cha B.-S., Choi J.-Y., Lee Y.-H. (2022). Ezetimibe combination therapy with statin for non-alcoholic fatty liver disease: An open-label randomized controlled trial (ESSENTIAL study). BMC Med..

[148] Crisby M., Nilsson J., Kostulas V., Björkhem I., Diczfalusy U. (1997). Localization of sterol 27-hydroxylase immuno-reactivity in human atherosclerotic plaques. Biochim. Biophys. Acta (BBA) Lipids Lipid Metab..

[149] Allen A.M., Taylor J.M.W., Graham A. (2013). Mitochondrial (dys)function and regulation of macrophage cholesterol efflux. Clin. Sci..

[150] Hofmann A.F., Hagey L.R. (2014). Key discoveries in bile acid chemistry and biology and their clinical applications: History of the last eight decades. J. Lipid Res..

